# Supplementary material for: Activation of the archaeal ion channel MthK is exquisitely regulated by temperature
Source: eLife. 2020 Dec 4;9:e59055. doi: 10.7554/eLife.59055 (PMC7717905; doi:10.7554/eLife.59055)
Supplement: Transparent reporting form [file elife-59055-transrepform.docx]

***eLife’s* transparent reporting form**

We encourage authors to provide detailed information *within their submission* to facilitate the interpretation and replication of experiments. Authors can upload supporting documentation to indicate the use of appropriate reporting guidelines for health-related research (see [EQUATOR Network](http://www.equator-network.org/%20)), life science research (see the [BioSharing Information Resource](https://biosharing.org/)), or the [ARRIVE guidelines](http://www.plosbiology.org/article/info:doi/10.1371/journal.pbio.1000412) for reporting work involving animal research. Where applicable, authors should refer to any relevant reporting standards documents in this form.

If you have any questions, please consult our Journal Policies and/or contact us: [editorial@elifesciences.org](mailto:editorial@elifesciences.org).

**Sample-size estimation**

- You should state whether an appropriate sample size was computed when the study was being designed
- You should state the statistical method of sample size computation and any required assumptions
- If no explicit power analysis was used, you should describe how you decided what sample (replicate) size (number) to use

Please outline where this information can be found within the submission (e.g., sections or figure legends), or explain why this information doesn’t apply to your submission:

Sample size estimation is not applicable. For all the data that has error bars shown in this study, a minimum repeat number of three was chosed.

**Replicates**

- You should report how often each experiment was performed
- You should include a definition of biological versus technical replication
- The data obtained should be provided and sufficient information should be provided to indicate the number of independent biological and/or technical replicates
- If you encountered any outliers, you should describe how these were handled
- Criteria for exclusion/inclusion of data should be clearly stated
- High-throughput sequence data should be uploaded before submission, with a private link for reviewers provided (these are available from both GEO and ArrayExpress)

Please outline where this information can be found within the submission (e.g., sections or figure legends), or explain why this information doesn’t apply to your submission:

For the mini suspension culture rescue assay shown in Figure 1C and Figure 1- figure supplement 1,

n=4 for MthK FL 37 °C with and without blocker;

n=3 for MthK FL 24 °C without blocker, n=4 for with blocker;

n=4 for MthK IR 37 °C and 24 °C, both with and without blocker;

n=3 for MthK ΔC 37 °C with and without blocker;

n=3 for MthK ΔC 24 °C without blocker, n=4 with blocker;

n=3 for empty vector at 37 °C both with and without blocker and

n=5 for empty vector at 24 °C both with and without blocker independent experiments.

The independent biological replicates are from different colonies of transformed bacterial cells.

For the macroscopic recordings from giant *E. coli* spheroplasts,

n = 4 independent measurements for 1.5 mM Calcium and

n = 3 independent measurements for 0.3 mM Barium.

The independent biological replicates are from inside-out patches of different spheroplasts.

For single channel recordings shown in Figure 2C and 2D, n = 10 (21 °C), 4 (26 °C), 4 (32 °C), 5 (37 °C) independent measurements. The independent replicates are patches from different proteoliposomes reconstituted in different batches, they should be viewed as biological replicates.

For single channel recordings shown in Figure 2-figure supplement 3,

n = 5 independent recordings. The independent replicates are different patches from proteoliposomes that are reconstituted in the same batch, they are technical replicates.

For single channel recordings shown in Figure 3C, n = 5 independent patches. The independent replicates are different patches from different proteoliposomes that are reconstituted in the same batch, they are technical replicates.

For single channel recordings shown in Figure 4C and Figure 4-figure supplement 1, n = 10 (0.1 mM Ca2+, 21 °C), 3 (0.2 mM Ca2+, 21 °C), 5 (0.5 mM Ca2+, 21 °C), 6 (1 mM Ca2+, 21 °C), 4 (2 mM Ca2+, 21 °C), 4 (5 mM Ca2+, 21 °C), 5 (10 mM Ca2+, 21 °C), 4 (0.1 mM Ca2+, 37 °C), 3 (0.2 mM Ca2+, 37 °C), 4 (0.5 mM Ca2+, 37 °C), 3 (1 mM Ca2+, 37 °C), 4 (2 mM Ca2+, 37 °C), 3 (5 mM Ca2+, 37 °C), and 3 (10 mM Ca2+, 37 °C) independent recordings. The independent replicates are different patches from proteoliposomes reconstituted in different batches, they should be viewed as biological replicates.

We used all the data for analysis except in one instance where the single channel recording at 0.1 mM Ca^2+^ at low temperature was excluded because the observed activity was two orders of magnitude lower than the mean obtained from 10 independent recordings.

The replicate numbers for each experiment are also indicated in the corresponding figure legends.

**Statistical reporting**

- Statistical analysis methods should be described and justified
- Raw data should be presented in figures whenever informative to do so (typically when N per group is less than 10)
- For each experiment, you should identify the statistical tests used, exact values of N, definitions of center, methods of multiple test correction, and dispersion and precision measures (e.g., mean, median, SD, SEM, confidence intervals; and, for the major substantive results, a measure of effect size (e.g., Pearson's r, Cohen's d)
- Report exact p-values wherever possible alongside the summary statistics and 95% confidence intervals. These should be reported for all key questions and not only when the p-value is less than 0.05.

Please outline where this information can be found within the submission (e.g., sections or figure legends), or explain why this information doesn’t apply to your submission:

All statistical tests were performed using OriginPro program. Data are presented as mean±SEM. Raw data were presented for the Po value in Figure 2 and Figure 2 supplement 3 as individual points, and box graph were used to represent data structure. Where applicable, student’s two sample T-test was used to evaluate the statistical significance of the results of two independently collected pools of data assuming non-equal variance. P > 0.05 was considered statistically non-significant; *, P ≤ 0.05; **, P ≤ 0.01.

(For large datasets, or papers with a very large number of statistical tests, you may upload a single table file with tests, Ns, etc., with reference to sections in the manuscript.)

**Group allocation**

- Indicate how samples were allocated into experimental groups (in the case of clinical studies, please specify allocation to treatment method); if randomization was used, please also state if restricted randomization was applied
- Indicate if masking was used during group allocation, data collection and/or data analysis

Please outline where this information can be found within the submission (e.g., sections or figure legends), or explain why this information doesn’t apply to your submission:

Not applicable.

**Additional data files (“source data”)**

- We encourage you to upload relevant additional data files, such as numerical data that are represented as a graph in a figure, or as a summary table
- Where provided, these should be in the most useful format, and they can be uploaded as “Source data” files linked to a main figure or table
- Include model definition files including the full list of parameters used
- Include code used for data analysis (e.g., R, MatLab)
- Avoid stating that data files are “available upon request”

Please indicate the figures or tables for which source data files have been provided:

All the main figures and supplemental figures have been supplemented with source data files.
